# Viral Hijacking of BET Proteins

**DOI:** 10.3390/v14102274

**Published:** 2022-10-17

**Authors:** Irene P. Chen, Melanie Ott

**Affiliations:** 1 Gladstone Institutes, San Francisco, CA 94158, USA; 2 Department of Medicine, University of California, San Francisco, San Francisco, CA 94143, USA; 3 Chan Zuckerberg Biohub, San Francisco, CA 94158, USA

**Keywords:** Coronavirus, Flavivirus, Papillomavirus, Polyomavirus, Retrovirus, Herpesvirus, Hepatitis B virus, BET proteins, BET inhibitors, RVX-208, JQ1, BRD4, host-pathogen interactions

## Abstract

Proteins of the bromodomain and exterminal domain (BET) family mediate critical host functions such as cell proliferation, transcriptional regulation, and the innate immune response, which makes them preferred targets for viruses. These multidomain proteins are best known as transcriptional effectors able to read acetylated histone and non-histone proteins through their tandem bromodomains. They also contain other short motif-binding domains such as the extraterminal domain, which recognizes transcriptional regulatory proteins. Here, we describe how different viruses have evolved to hijack or disrupt host BET protein function through direct interactions with BET family members to support their own propagation. The network of virus-BET interactions emerges as highly intricate, which may complicate the use of small-molecule BET inhibitors–currently in clinical development for the treatment of cancer and cardiovascular diseases–to treat viral infections.

## 1. Introduction

The bromodomain and extraterminal domain (BET) proteins, consisting of BRD2, BRD3, BRD4, and BRDT, are characterized by their ability to bind acetylated lysines. BRD2, BRD3, and BRD4 are expressed ubiquitously and will be the focus of this review, while BRDT expression is restricted to the male germline. BET proteins are major transcriptional regulators and when dysregulated, are implicated in cancer as well as autoimmune, cardiovascular, and metabolic diseases [1]. Consequently, there is intense focus on finding selective and potent BET inhibitors to restore normal gene regulation for the treatment of these conditions.

In recent years, an increasing number of viruses have been found to directly engage with BET proteins for their survival. The wide variety of fundamental roles BET proteins play in cells, combined with their shared multifunctional domains, make them natural targets of viruses. Below, we describe general functions of each BET protein and summarize how different viruses directly hijack or subvert BET proteins for their own benefit.

## 2. Domain Architecture of BET Proteins

**Figure 1:**
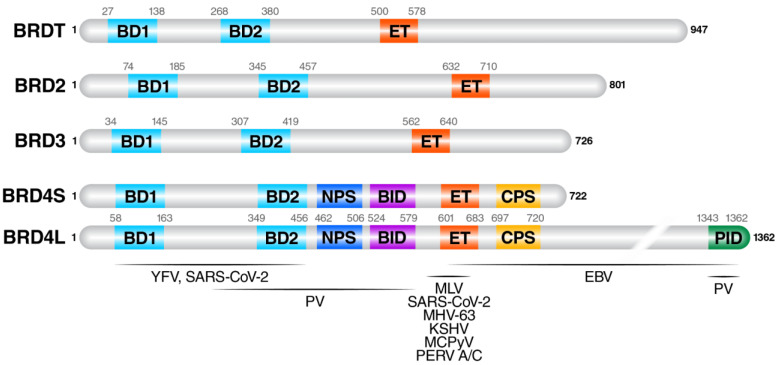
BET protein family members. BRDT, BRD2, BRD3, and BRD4 are the four mammalian members of this family. The main functional domains and their amino acid positions are indicated: BD1 (bromodomain 1), BD2 (bromodomain 2), ET (extraterminal domain), PID (P-TEFb interacting domain), BID (basic residue-enriched domain), NPS (N-terminal phosphorylation site), and CPS (C-terminal phosphorylation site). Horizontal lines span the regions of BRD4 that directly interact with the viruses described in this review; note however that other BET proteins can also interact with viruses. See Table 1 for more information.

The functions of BET proteins have been widely associated with their conserved domains reflected in their name: bromodomains (BDs) and extraterminal (ET) domain (Figure 1). BET proteins have two tandem, N-terminal BDs (BD1 and BD2) that engage peptides containing acetylated lysine residues optimally spaced by two amino acids (Kac-XX-Kac) [2,3,4]. Through their BDs, BET proteins act as transcriptional regulators by interacting with acetylated histones, transcription factors, and viral proteins [2,3,5]. The extraterminal domain mediates protein-protein interactions and recognizes a short peptide motif, “KIKL,” conserved across many different transcriptional regulators, including catalytic subunits of chromatin remodeling complexes [6]. Other smaller domains identified within BET proteins include a basic interacting domain (BID), phosphorylation-dependent interacting domain (PDID), and N- and C-terminal serine-rich domains that can be phosphorylated (NPS and CPS) and mediate more protein-protein interactions and auto-regulation of function (Figure 1) [7,8,9,10,11]. BET proteins can also form hetero- or homodimers within the family through a shared motif B that is required for BET protein association to chromatin [12].

## 3. Overlapping and Distinct Functions of BET Proteins

While BET proteins share many conserved protein domains and often interact with the same proteins, they can perform unique functions (as reviewed in [13]), in part because of a wide range of binding affinities for the same targets. Lower sequence conservation outside the BD and ET domains may also enable distinct functions and interactions for each BET family member. The functions mentioned below pertain to BET protein-mediated processes that viruses directly exploit as opposed to immunomodulatory functions that BET proteins transcriptionally control during disease as previously reviewed by others (reviewed in [14]).

### 3.1. BRD2

BRD2 was originally identified as a mitogen-stimulated nuclear kinase [15]. Upon serum stimulation, it binds the promoters of cell cycle genes and regulates E2F2-dependent genes required for the G1/S transition of the cell cycle [16,17]. BRD2, along with BRD3, has intrinsic histone chaperone activity that allows RNA Polymerase II (Pol II) to process through hyperacetylated nucleosomes [18]. 

BRD2 also regulates transcription by serving as a scaffold for proteins to associate with chromatin. It has been found in association with five major chromatin-related complexes: (1) Pol II, the core promoter, and TATA binding factor (TBP)-associated factors (TAFs), (2) activated transcription factors E2F and DP-1, (3) Mediator complex subunits, (4) histone modification enzymes (HDAC11 and CBP/p300), and (5) SWI/SNF remodeling complex subunits [19]. Depending on its chromatin-binding partners, BRD2 either activates transcription (when associated with Mediator complex and TAFs) or represses it (when associated with HDAC11 and the SWI/SNF chromatin-remodeling complexes). Beyond this, BRD2 can influence transcription by regulating higher-order chromatin architecture. BRD2 is recruited to heterochromatin boundaries and protects euchromatic histone modifications, thus strengthening boundaries and limiting the spread of heterochromatin [20]. It also colocalizes with chromatin architecture/insulator protein CCCTC-binding factor (CTCF) to form transcriptional boundaries and restrict aberrant expression of genes flanking such boundaries [21].

### 3.2. BRD3

BRD3, like other BET proteins, is able to bind acetylated histones and transcription factors to regulate transcription. It shares many functions with BRD2, such as regulating cyclin D1 expression and facilitating the elongation of transcripts by Pol II through hyperacetylated nucleosomes [18,22,23]. In concert with BRD2, it binds directly to the transcription factor GATA1 to synergistically regulate GATA1-mediated erythroid gene expression [24]. Additionally, BRD3 is able to interact with RNA and transcription factors to form phase-separated condensates on enhancers as a transcriptional regulatory strategy [25].

### 3.3. BRD4

BRD4 is the best characterized member of the family and has prominent roles in cell proliferation and transcription. It is one of the few nuclear factors retained in the nucleus, bound exclusively to chromosomes, during the M phase, when most factors are dissociated in response to global arrest of transcription. As a mitotic bookmark, BRD4 marks the transcription start sites (TSSs) of many genes programmed to express during or immediately after mitosis, and promotes transition into S phase [26,27]. Beyond its role in cell proliferation, it is able initiate transcription elongation by recruiting positive transcription elongation factor (P-TEFb) through a P-TEFb interacting domain (PID) found in the long isoform of BRD4 (BRD4L). The binding of BRD4L to active P-TEFb prevents P-TEFb’s interaction with inhibitory factors and facilitates Pol II pause release [28,29]. BRD4 can also recruit transcription factors to promoters, interact with catalytic enzymes of the NuRD and SWI/SNF chromatin remodelers, and with lysine methyltransferases, to loosen and compact chromatin for controlling transcription [9,14,30,31]. BRD4 also has atypical kinase activity that allows it to phosphorylate Pol II CTD and facilitate transcription elongation independently of P-TEFb [29,32]. Additionally, BRD4 has intrinsic histone acetyltransferase (HAT) activity where it mediates chromatin decompaction by acetylating and evicting nucleosomes of target genes, thus activating their transcription [32]. Post translational modifications on BRD4, such as phosphorylation of NPS by CK2, methylation of BD1 by SETD6, proline hydroxylation by PDH2, and ubiquitination by SPOP, modulate BRD4’s function in chromatin localization, transcription factor recruitment, protein stability, and transcriptional activation [10,13,33,34,35].

On the other hand, the short isoform of BRD4 (BRD4S) lacks PID and intrinsic HAT activity, but shares the same BD and ET domains as BRD4L. An unique aspect of BRD4S is that it plays an active role in repairing DNA double-strand breaks (DSBs) marked by hyperacetylation of histone H4 and phosphorylation of H2AX. BRD4S is typically recruited to DSBs, where it forms DNA repair complexes by recruiting repair proteins [36,37]. Additionally, BRD4S can act as a chromatin insulator regulating responses to DNA damage [38]. In this role, BRD4S inhibits the DNA damage response (DDR) by recruiting the condensin II chromatin remodeling complex to acetylated histones via its BDs to limit chromatin accessibility to repair proteins. 

## 4. Current BET Inhibitors Target All Family Members

Because BET proteins play many vital functions, their dysregulation underlies many diseases. One way to restore normal cellular function is to disrupt BET proteins’ ability to alter gene expression in diseased cells. All current BET inhibitors are small molecules that compete for binding of BDs to their natural ligand, acetyllysines (more extensively reviewed in [39]). Most inhibitors, such as JQ1, IBET-151 and OTX015, non covalently bind BDs of all BET proteins as they are highly conserved [40,41,42]. Small differences in amino acids, polarity, and hydrophobicity in BD binding pockets have allowed the recent development of BD1- and BD2-specific inhibitors (Olinone and GSK-778 for BD1; Apabetalone [RVX-208] and ABBV-744 for BD2), but they still target all BET proteins indiscriminately [4,40,43,44,45,46,47,48,49]. Proteolytic targeting chimeric (PROTAC) compounds (dBET6 and ARV-825) that induce BET protein degradation are also being investigated [50,51,52]. Most BET inhibitors are currently being tested for various cancer and cardiovascular diseases, with growing potential for applications in viral diseases.

## 5. The Role of BET Proteins in the Viral Life Cycle

**Figure 2:**
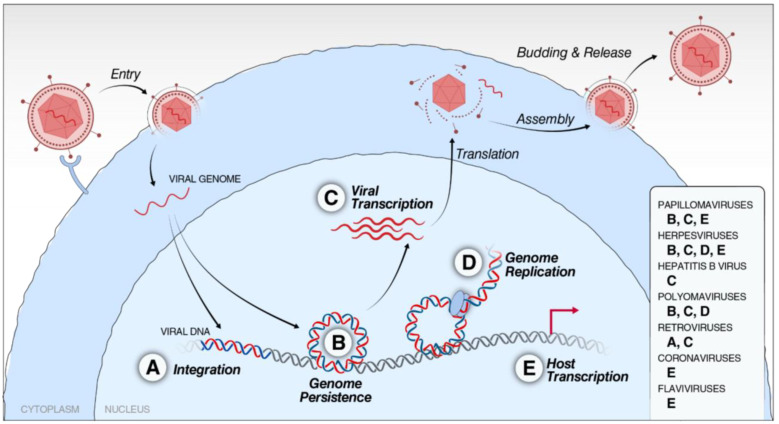
Steps in a generic viral life cycle where BET proteins are involved. Many viruses interact with BET family members to mediate viral processes (A–D) and disrupt cellular transcription for their own benefit (E). (A) Integration site selection of MLV, a gammaretrovirus, is facilitated by an interaction between its viral IN protein and BET proteins, leading to integration into genomic regions associated with BET proteins. (B) PV, herpesviruses, and polyomaviruses take advantage of the association of BET proteins to host chromosomes to maintain their viral genomes in infected cells. (C) Certain herpesviruses (EBV and HSV), PV, HBV, and polyomaviruses recruit the BRD4:P-TEFb complex to facilitate viral transcription, while retroviruses encode viral proteins that directly bind the PID of BRD4 to redirect host transcriptional machinery to the viral genes. (D) Herpesviruses and polyomaviruses recruit BRD4 along with other host replication machinery, such as RFC or DNA polymerase δ, facilitate genome replication at their replication foci. (E) PV, Herpesviruses, SARS-CoV-2, and flaviviruses interact with BET proteins to disrupt homeostatic gene transcriptional programs controlled by BET proteins during infection to generate a cell state more favorable to viral replication and virus-induced carcinogenesis.

This review focuses on direct interactions between viral proteins or genome with members of the BET family during different steps of the viral life cycle (Figure 2).

### 5.1. Coordinators of Viral Genome Integration

A hallmark of retroviral replication is the integration into the host genome. Interestingly, the site of retroviral integration is not at all random; most retroviruses are guided to certain regions of the host genome by its integrase (IN) protein via interactions with specific host proteins. Murine leukemia virus (MLV), a gammaretrovirus, preferentially integrates near transcription start sites (TSSs), CpG islands, and DNase I-hypersensitive sites, which correlate with BET protein-chromatin binding sites, and are best predicted by BRD2 distribution [53,54,55,56]. MLV integration site selection is guided by interactions between its IN and the ET domains of BET proteins [54,55]. When cells were infected with MLV in the presence of the BET inhibitors, the number of integrated MLV copies significantly decreased and integration was redirected away from TSSs [54,55]. Conversely, overexpression of the BRD2 ET domain increased the number of integrated MLV genomes [54]. Moreover, the ET domains of BRD2 and BRD4 bind the INs of other gammaretroviruses (but not alpha-, beta-, and delta-retroviruses, or lentiviruses), including porcine endogenous retrovirus-A/C (PERV A/C), feline leukemia virus (FeLV), and avian Reticulo-Endotheliosis Virus (REV) in a similar manner, suggesting that gammaretroviruses have evolved a common association with BET proteins to select their integration sites [54,57,58].

### 5.2. Drivers of Viral Genome Persistence

Unlike many viruses that cause the lysis of the infected host cells, papillomaviruses (PVs) and herpesviruses persist at relatively low copy numbers without destroying their host cells. Both PVs and herpesviruses maintain their DNA genomes as episomes tethered to chromatin via BET proteins. This allows their viral genomes to be partitioned along with the host chromosomes into daughter cells during mitosis, a process termed mitotic segregation. 

PV episomes tether to host chromosomes via the interaction between its E2 protein and the C-terminus of chromatin-associated BRD4 to ensure that its genome is retained in the nucleus and partitioned into daughter cells [9,59,60,61]. In fact, the E2:BRD4 complexes are visible as distinct dots on the mitotic chromosome after successful infection [62,63,64]. The overexpression of just the BRD4 C-terminus dominantly prevents E2 from binding chromosomes and causes the loss of PV genomes in cells [64,65,66]. While E2s of all PVs interact with BRD4, not all E2 proteins are readily observed to bind mitotic chromosomes because of differences in their affinity for BRD4 [67,68]. E2 proteins from BPV, HPV, and cottontail rabbit PV (CRPV) bind tightly to BRD4 whereas alpha- and beta-PVs bind only weakly [62,64,67,69,70]. This suggests that not all PVs maintain their genomes through a BRD4-dependent mechanism.

Similar to PV’s E2, the LANA protein of Kaposi’s sarcoma associated herpesvirus (KSHV) likely maintains KSHV’s episome through BRD2 and BRD4. LANA contacts the ET domains of BET proteins [6,71]. Typically, BRD2 is associated with euchromatin, but it relocalizes to heterochromatic regions in the presence of LANA and colocalizes with LANA in infected mitotic and interphase cells [72,73]. Given the role of BRD2 in maintaining transcriptional boundaries and spreading histone acetylation, the LANA:BRD2 complex is proposed to maintain viral genome persistence by mediating local euchromatin formation around the episome, thus tethering it to heterochromatin [20]. BRD2 also stabilizes and possibly maintains mitotic segregation of another herpesvirus, Epstein-Barr virus (EBV) [74]. Reliance on BET proteins to maintain viral genomes is further found in the unrelated raccoon polyomavirus (RacPyV), where treating infected cells with JQ1 reduces viral genome copies [75]. Overall, many DNA viruses take advantage of the fact that BET proteins stably bind chromatin to persist in host cells long-term.

### 5.3. Organizers of Viral Genome Replication

BET proteins facilitate the replication of oncogenic DNA viruses such as polyomavirus, human papillomaviruses (HPV), and EBV. Merkel Cell polyomavirus (MCPyV) recruits BRD4 via its large T (LT) antigen, a protein that binds to the viral origin of replication (Ori) and functions as a helicase for unwinding viral DNA for replication [76]. Normally diffused in the nucleus, BRD4 forms distinct nuclear foci in the presence of both Ori and LT, suggesting Ori traffics the viral replication complex to specific nuclear locations [77]. The interaction is mediated by the ET domain of BRD4 and results in BRD4-mediated recruitment of cellular replication complex C (RFC), essential for DNA replication, to LT/Ori foci. Viral DNA replication is reduced upon BRD4 knockdown, but rescued in a dose-dependent manner upon BRD4 reconstitution [77]. Treating infected cells with JQ1 increased viral DNA replication but not viral transcription, most likely because it released chromatin-bound BRD4 and allowed the assembly of more MCPyV LT/Ori replication complexes [77]. The genome replication of a related polyomavirus, John Cunningham polyomavirus (JCPyV) is similarly reduced in the presence of JQ1 [78].

BRD4 is similarly involved in two different steps of the PV genome replication process. HPVs activate DDR and position DDR factors, such as DNA polymerase δ, on the HPV genome for replication [59,68,79]. Given that BRD4 acts as an important scaffolding protein in DDR, it is speculated to be involved in recruiting the replication machinery to the HPV genome [80,81]. Additionally, BRD4 is recruited to active HPV replication origin foci, along with HPV’s E2 protein and cellular RFC and DNA polymerase [82]. The E2:BRD4 interaction is dependent on phosphorylation status of both E2 and NPS and PDID in the C terminus of BRD4 [9,10,83]. Mutagenesis disrupting the E2:BRD4 interaction abolishes the formation of HPV replication complexes and impairs viral DNA replication [84]. The addition of JQ1 also enhances HPV genome replication and is thought to function in a manner similar to its effect on MCPyV [82]. In contrast, BET inhibitors block EBV DNA replication initiation by preventing BET proteins from localizing at the lytic origins of replication (OriLyt) [85].

### 5.4. Accomplices in Viral Transcription

As BET proteins are well-known cellular transcriptional regulators, it is not surprising that viruses subvert their function for transcribing their own genome. PVs, EBV, hepatitis B virus (HBV), herpes simplex virus (HSV), and JCPyV rely on BRD4:P-TEFb for viral transcription, whereas human cytomegalovirus (HCMV) and retroviruses compete with BRD4 for binding to P-TEFb.

#### 5.4.1. Recruitment of the BRD4:P-TEFb Complex

The E2 protein of PVs can both activate and repress viral transcription, depending on where it binds in relation to promoter elements [65,86,87,88,89]. In both cases, it depends on BRD4: disrupting E2:BRD4 with a dominant-negative BRD4 C-terminal peptide blocks the transactivation function of many PV E2 proteins; conversely, BRD4 knockdown reduces E2’s ability to repress genes that regulate the transition between lytic and latent infection [67,90,91,92]. Additionally, Epstein-Barr nuclear antigen 1 (EBNA1) interacts with the C-terminal domain of BRD4, and this interaction is important for EBNA1-mediated viral transcription from the family of repeats (FR) enhancer elements in the EBV genome [74]. Inhibiting BRD4 with JQ1 prevented the expression of viral immediate-early and late viral proteins during EBV reactivation [85].

Furthermore, different P-TEFb-containing complexes, including BRD4 or superelongation complex (SEC), have been reported to bind the genome of HBV [93]. JQ1 treatment enhances BRD4 occupancy on the viral genome and induces HBV transcription, suggesting that BRD4 does not rely on BDs to regulate viral transcription [93]. JQ1 most likely releases chromatin-bound BRD4 to allow the recruitment of BRD4:P-TEFb to the viral genome, a novel mechanism in which HBV hijacks host P-TEFb complexes for its own transcription. This effect has important clinical implications, as BET inhibitors used to treat infections or other conditions could inadvertently reactivate latent HBV.

BET inhibitors are also investigated as a means to dysregulate HSV gene expression (further reviewed in [94]). BET inhibitors increase viral transcripts levels, protein production, and infectious virion production, resulting in HSV reactivation in sensory ganglia explants and mouse models by increasing the recruitment of BRD4:P-TEFb to its viral promoters [95,96]. Unexpectedly, RVX-208, a BD2-specific inhibitor, had no effect on HSV replication suggesting BET inhibitor-mediated HSV reaction is primarily through BD1 [95].

JCPyV also relies on BRD4 to regulate viral transcription. The genome of JCPyV is divided into two transcriptional regions, early and late, by a noncoding regulatory region (NCRR) that contain many transcription factor binding sites, including an NF-κB binding site. BRD4 activates early JCPyV transcription through its involvement with recruiting NF-κB to the nucleus and its ability to coactivate viral gene transcription by binding to acetylated NF-κB P65 and recruiting P-TEFb [7,78]. JCPyV viral transcription is enhanced by ectopic expression of BRD4 and reduced upon BET inhibition with JQ1 [78].

#### 5.4.2. Competitive Binding to P-TEFb

Although no direct interaction between HCMV and BET proteins has been demonstrated, BRD4 is inextricably linked to HCMV latency and reactivation [97,98]. BRD4 colocalizes on the viral genome with HCMV’s immediate-early 2 protein (IE2) to initiate viral transcription [98]. BRD4’s ability to bind P-TEFb sequesters the transcriptional activator complex away from HCMV promoters and enforces latency. In the presence of BET inhibitors, P-TEFb is released from BRD4 and recruited to viral promoters by the SEC to induce viral gene expression [97]. Interestingly, BET inhibitor-mediated HCMV reactivation selectively induces expression of a limited subset of viral proteins that can trigger cytotoxic cell killing of latently infected cells without inducing viral DNA replication [97]. Thus, BET inhibitors have been proposed as a strategy to purge the latent HCMV reservoir.

Retroviruses such as human T-lymphotropic virus type 1 (HTLV-1) and human immunodeficiency virus type 1 (HIV-1) encode viral proteins that directly bind P-TEFb to sequester it for viral transcription. The Tax protein of HTLV-1 competes with BRD4L for binding the cyclin T1 subunit of P-TEFb [99]. Similar to BRD4L overexpression, Tax overexpression increases Pol II CTD phosphorylation, indicating that Tax is a positive regulator of P-TEFb. When BRD4L is overexpressed, Tax is no longer able to transactive the LTR promoter [99]. Similarly, the Tat protein of HIV-1 recruits P-TEFb to stimulate viral transcription elongation at HIV-1 promoters [28,100,101,102,103,104]. Both Tax and Tat competitively bind P-TEFb as a mechanism to decrease the number of BRD4:P-TEFb complexes and redirect the host transcriptional machinery to viral genes. Interestingly, BRD4L can also activate P-TEFb to positively regulate basal HIV-1 transcription in a Tat-independent manner [105]. The release of P-TEFb bound to BRD4L explains how BET inhibitors are able to reactivate HIV-1 from latency [106].

On the other hand, BRD4S is a Tat-independent repressor of HIV-1 transcription. BRD4S recruits BRG1, the catalytic subunit of the repressive BAF chromatin remodeling complex, to the LTR to repress HIV-1 transcription [107]. Specific knockdown of BRD4S alone can reactivate HIV-1 [107]. Strikingly, BRD4S and BRG1 share extensive overlap in chromatin occupancies genome-wide, notably enriched at class I (LINEs, SINEs, and LTRs) and II (DNA) transposon sequences [107]. The fact that BRD4S:BRG1 is enriched at endogenous LTR-containing sequences evolutionarily related to HIV-1 suggests that endogenous retroviral sequences share a common mechanism in recruiting the repressive function of this complex.

### 5.5. Unwilling Disruptors of Host Transcription

During infection, the cellular transcriptome is dramatically reprogrammed. Cells are not only inundated with viral transcripts, but also shifting from maintaining homeostatic processes to producing antiviral and inflammatory mediators or shutting off cellular transcription to control viral infection. Inevitably, viruses have evolved to manipulate the host transcriptome to create a cell state that favors viral replication and spread.

The strategy employed by yellow fever virus (YFV) and SARS-CoV-2 to modulate host transcription is histone mimicry. Both YFV capsid and SARS-CoV-2 envelope (E) proteins contain a histone H4- or H3-like motif, respectively, with two lysine residues that can interact with BDs of BET protein when acetylated [108,109,110]. Interestingly, the loss of YVF capsid:BRD4 interaction results in lower YFV infectivity and viral spread, suggesting the interaction optimizes the cell state for virion assembly and cell-to-cell spread [108]. The SARS-CoV-2 E:BRD4 interaction disrupts the function of BRD4 as a critical epigenetic regulator of innate immune genes [109]. E protein sequesters BRD4 and suppresses the expression of BRD4-regulated innate immune genes. The therapeutic use of BET inhibitors during infection further enhances viral replication and infectious particle production [109]. Additionally, the ET domain of BRD4 is predicted to bind a “K-X-K-X” motif in E protein in a BD-independent manner [110]. Other viruses known to manipulate the host’s transcription to their advantage include gammaherpesvirus 68 (MHV-68), whose orf73 interacts with the ET domains of BET proteins to activate cell cycle genes cyclin D2, D1 and E, and PV, whose E2 protein’s interaction with BRD4 regulates matrix metalloproteinase-9 (MMP-9) and c-Fos gene expression to create a cell state that is more favorable to PV-induced carcinogenesis [59,111,112].

## 6. Conclusions

The shared domains of BET proteins with defined interaction motifs allow viruses to easily engage with multiple members of the family, effectively targeting specific functions of individual BET proteins at the same time. Several major modes of engagement across different viruses emerge: (1) YFV and SARS-CoV-2 proteins have acetylated lysine residues that mimic acetylated histones to dissociate BET proteins via BDs from chromatin. (2) Herpesvirus and polyomavirus engage ET domains for viral genome maintenance and MLV for integration site selection. (3) Many viruses exploit BRD4’s ability to recruit the transcription complex P-TEFb not only for their own transcription, but also to modulate the host transcriptome and create a cell state favorable for infection and spread (Figure 2).

There is growing interest in using BET inhibitors as therapeutics for viral infection. They have been best studied in the context of HIV-1 eradication, where they act as latency reversing agents (LRAs) (further reviewed in [113]). The first BET inhibitor, JQ1, was a promising LRA in vitro and ex vivo, but is not a clinical candidate due to unfavorable pharmacokinetic properties [106,114,115,116]. Newer inhibitors, such as RXV-208, PF-1, and OTX-015, that demonstrate similar LRA potency in addition to having favorable oral bioavailability, fewer side effects, and synergistic effects with other LRAs, have yet to be evaluated in vivo [117,118,119,120]. BET inhibitors are also possible LRAs for latent HCMV and HSV infections and can limit growth of PV-, HCMV-, EBV-, and MCPyV-associated malignancies and diseases [64,77,85,94,95,121]. A caveat is that the use of BET inhibitors for non-viral indications in patients who harbor latent viruses (herpesviruses, HBV, or retroviruses) could result in viral reactivation and undesirable outcomes.

The intricacies of BET proteins’ functions in host transcriptional processes further complicate the use of pan-BET inhibitors. For example, BRD2 is a positive transcriptional regulator of SARS-CoV-2’s entry receptor, angiotensin converting enzyme 2 (ACE2). When given prophylactically, BET inhibitors can reduce ACE2 expression to limit viral infection [109,110,122,123,124,125,126]. But the time point of application is critical as viral infection in cell lines and mortality of infected mice are exacerbated when BET inhibitors are given at the time of infection, due to their immuno-suppressive effects [109]. However, later in infection, the immuno-suppressive effect of BET inhibitors may be desirable as it could block cytokine production associated with COVID-19 cardiac dysfunction [123]. Currently, RVX-208 is under clinical investigation for COVID-19 (clinicaltrials.gov, NCT04894266). A very detailed clinical treatment protocol is needed to avoid negative effects of the drugs and take full advantage of the opportunities offered by BET inhibitors. Selective BET inhibitors may alleviate the issue as BRD2 is primarily involved in ACE2 downregulation, a function that could be selectively targeted.

Another solution may be offered by compounds that selectively target one bromodomain over the other. For example, BD2-specific inhibition preferentially blocks enhancer-driven, stimulus-mediated induction of gene expression programs without affecting pre-existing transcriptional programs, an advantage for diseases like COVID-19, where inflammatory or pro-fibrotic signaling are activated *de novo* [47]. By contrast, pan- or BD1-specific inhibitors are more effective in disease states where extensive alteration of cellular programming occurs, such as cell proliferation and survival in cancer [47,127]. Furthermore, investigations into selectively targeting the phosphorylation sites of BRD4 offer a unique opportunity to specifically inhibit transcriptional programs mediated by PDID and NPS, such as BRD4:HPV E2, without disrupting broad chromatin-binding activity of BRD4 [128,129]. The differential actions of specific domains in BET proteins may prove to be pivotal for better refinement of inhibitors and their therapeutic applications in viral diseases.

**Table 1:** BET protein interactions with viruses. Parentheses indicate punitive interaction domains. * indicates viral genome regions. ** indicates indirect interaction, but competition for binding to P-TEFb.

Virus	Viral Protein/Genome	BET Protein	BET Domain	Functions	References
MLV	IN	BRD2, BRD3, BRD4	ET	Integration site selection	[54,55,56]
PERV A/C	IN	BRD2, BRD3, BRD4	ET	Integration cofactor	[57]
Papillomavirus (HPV, CRPV, BPV)	E2	BRD4	C-terminus, NPS, BID	E2 stability, E2-mediated viral and host transcription, tethering viral genome to host chromatin	[9,59,63,64,65,66]
KSHV	LANA	BRD2, BRD3, BRD4	ET	Tethering viral genome to host chromatin, LANA-mediated viral and host transcription	[6,71,130]
MHV-68	orf73	BRD2, BRD3, BRD4	ET	Tethering viral genome to chromatin, orf73-mediated host transcription	[112]
EBV	EBNA1 OriLyt *	BRD2, BRD3, BRD4	(C-terminus)	EBNA1-mediated viral transcription, tethering viral genome to host chromatin	[74,85]
HCMV	HCMV promoters *	BRD4	PID	Competence for P-TEFb	[97,98]
MCPyV	LT	BRD4	ET	Viral genome replication	[77]
JCPyV	NCRR *	BRD4	(BDs, PID)	Viral transcription and genome replication	[78]
RacPyV	RacPyV genome *	BRD4	(BDs, PID)	Viral transcription and genome replication, tether genome to host chromatin	[75]
HBV	HBV genome *	BRD4	BD-independent	Viral transcription	[93]
HTLV-1	Tax **	BRD4	PID	Competence for P-TEFb	[57,99]
HIV	Tat ** LTR *	BRD4	PID BDs	Competence for P-TEFb Tat-mediated transcription, enforce viral latency	[103,105,106,107,131]
YFV	Capsid	BRD2, BRD3, BRD4	BDs	Host transcription	[108]
SARS-CoV-2	E	BRD2, BRD4	BDs, ET	Host transcription	[109,110]

Despite growing interest and an impressive list of reports detailing virus-BET interactions, many open questions remain. For example, since viral proteins often only engage with a particular domain of a BET protein, can other domains normally engage with regular partners and still fulfill their functions? While many viral proteins are able to interact with more than one BET protein, most studies primarily focus on BRD4 as it is best understood. What consequences of the same interaction with BRD2 or BRD3 are we then overlooking? Despite these unknowns, our current understanding of virus-BET protein interactions underscore the importance of BET proteins in viral infection. Continued scrutiny of the BET proteins will yield a better understanding of the impact of viruses on their hosts and vice versa. It will also reveal the full picture of pro- and anti-viral functions of BET proteins and allow effective application of BET inhibitors to fight viral infections. 

## Data Availability

Not applicable.
